# The effect of supplementation with rubber seed kernel pellet on in vitro rumen fermentation characteristics and fatty acid profiles in swamp buffalo

**DOI:** 10.1186/s12917-024-04017-8

**Published:** 2024-05-06

**Authors:** Nirawan Gunun, Chatchai Kaewpila, Waroon Khota, Thachawech Kimprasit, Anusorn Cherdthong, Pongsatorn Gunun

**Affiliations:** 1 Department of Animal Science, Faculty of Technology, Udon Thani Rajabhat University, Udon Thani, 41000 Thailand; 2 Department of Animal Science, Faculty of Natural Resources, Rajamangala University of Technology Isan, Sakon Nakhon Campus, Sakon Nakhon, 47160 Thailand; 3 Chakri Naruebodindra Medical Institute, Faculty of Medicine Ramathibodi Hospital, Mahidol Univerisity, Samut Prakan, 10540 Thailand; 4 Tropical Feed Resources Research and Development Center (TROFREC), Department of Animal Science, Faculty of Agriculture, Khon Kaen University, Khon Kaen, 40002 Thailand

**Keywords:** Rubber seed kernel pellet, Unsaturated fatty acids, Biohydrogenation, Rumen fermentation, In vitro

## Abstract

**Background:**

Rubber seed kernel is a by-product derived from rubber tree plantations. It is rich in C18 unsaturated fatty acids (UFA) and has the potential to be used as a protein source for ruminant diets. This investigation has been conducted to determine the influence of rubber seed kernel pellet (RUSKEP) supplementation on in vitro rumen fermentation characteristics and fatty acid profiles in swamp buffalo. Using a completely randomized design (CRD) and supplementation of RUSKEP at 0, 2, 4, 6, 8, and 10% dry matter (DM) of substrate.

**Results:**

The supplementation with RUSKEP had no effect on gas kinetics, cumulative gas production, or degradability. Ruminal pH decreased linearly (*P* < 0.01) and ammonia-nitrogen (NH_3_-N) concentration decreased quadratically (*P* < 0.01) by RUSKEP supplementation. The proportion of acetate (C2) decreased linearly (*P* < 0.01), but propionate (C3) and butyrate (C4) increased linearly (*P* < 0.01), resulting in a decrease in the acetate to propionate ratio (C2:C3) (*P* < 0.01) by RUSKEP supplementation. With an increasing level of dietary RUSKEP, there was a slight increase in UFA in the rumen by increasing the oleic acid (OA; C18:1 cis-9 + trans-9), linoleic acid (LA; C18:2 cis-9,12 + trans-9,12), and α-linolenic acid (ALA; C18:3 cis-9,12,15) concentrations (*P* < 0.01).

**Conclusions:**

Adding up to 10% of RUSKEP could improve in vitro rumen fermentation and C18 unsaturated fatty acids, especially ALA, in swamp buffalo.

## Introduction

Animals currently provide the best sources of high-quality, nutritious food for humans. Consumers demand foods with a healthier fatty acid profile that have lower saturated fatty acids (SFA) and higher UFA, including mono-unsaturated fatty acids (MUFA) and poly-unsaturated fatty acids (PUFA) [1]. PUFA, especially omega-6 and omega-3, are essential to human health [2]. The diet composition of ruminants has the most significant impact on the fatty acid composition of meat and milk [3]. The ruminant diet (forages, oilseeds, and by-products) has higher levels of UFA, mostly LA (omega-6, ω6) and ALA (omega-3, ω3), while some feedstuffs have high MUFA, particularly OA (omega-9, ω9) [4, 5]. Nevertheless, dietary UFA can be toxic to rumen microbes because it damages cell integrity [6]. Therefore, following hydrolysis, rumen microorganisms convert UFA to SFA via a process known as biohydrogenation [7]. In regards to the potential improvements to human health, a number of studies have examined the effects of adding oilseeds high in UFA to the diets of ruminants [8, 9].

The rubber tree (*Hevea brasiliensis*) has been grown mostly in Southeast Asia, and its by-product, the rubber seed, consists of shell and kernel [10]. Rubber seed kernel (RSK) contains 18.6–22.2% CP, 33.4–39.4% ether extract (EE), and 6,680–8,000 kcal/kg DM gross energy (GE) [11, 12]. The RSK has been identified as a source of UFA because it contained 40.0% LA, 21.2% ALA, and 22.5% OA [12]. Hence, these by-products have potential use as supplementary protein and fat sources for ruminants. In earlier research, the addition of yeast-fermented RSK at 25% in concentrate diets did not change feed utilization, rumen fermentation, or microbial protein synthesis in dairy heifers [10]. According to Gunun et al. [11], adding yeast-fermented RSK concentrate as a protein source didn’t have any negative effects on feed intake, nutrient digestibility, rumen fermentation, hematology, microbial protein synthesis, or milk yield. However, dairy cows’ milk fat and total solids are slightly lower. Using the in vitro technique, Gunun et al. [12] reported that heating RSK in a hot air oven inhibited the biohydrogenation of ruminal UFA, especially ALA and OA. Pi et al. [13, 14] found that adding 4% rubber seed oil to the diet of dairy cows improved digestibility, rumen fermentation, and UFA in the rumen and milk, especially vaccenic acid (VA; C18:1 trans-11), conjugated linoleic acid (CLA; C18:2 cis-9, trans-11), and ALA.

The feed industry for animals primarily uses pelleting as a thermal processing technology [15]. The various positive effects of feed pellets include higher nutrition density, feed selection prevention, improved feed utilization, and rumen fermentation characteristics, resulting in increased productivity of ruminants [16, 17]. Additionally, pellets are simple to transport and store. However, there is no research into the effects of different levels of RSK-based pellet products in ruminants on rumen fermentation and biohydrogenation. Thus, our hypothesis is that supplementation with RUSKEP may enhance rumen fermentation patterns and fatty acid compositions, particularly C18 UFA. Therefore, the objective of this research was to evaluate the impact of supplementation with RUSKEP on in vitro fermentation characteristics and the biohydrogenation of C18 UFA in rumen fluid from swamp buffalo.

## Results

### Chemical composition of diets

The concentrate diets and rice straw had CP levels of 14.6% and 3.9%, respectively (Table 1). The RUSKEP consists of 19.8% of CP, 27.3% of EE, and 8,162.2 kcal/kg DM of GE (Table 2). In RUSKEP, the OA, LA, and ALA content amount to 21.2%, 49.7%, and 13.4%, respectively.

**Table 1:** Ingredients and chemical composition of the diet used in the experiment

Item	Concentrate	Rice straw
Ingredient, kg DM		
Cassava chip	45.0	-
Rice bran	19.0	-
Dried brewers’ grains	17.5	-
Soybean meal	14.0	-
Molasses	2.0	-
Mineral and vitamin mixture^1^	1.0	-
Urea	0.5	-
Salt	0.5	-
Sulfur	0.5	-
Chemical composition		
Dry matter, %	87.9	91.0
Organic matter, %DM	88.9	89.8
Crude protein, %DM	14.6	3.9
Ether extract, %DM	1.8	0.6
Neutral detergent fiber, %DM	39.9	70.6
Acid detergent fiber, %DM	21.4	43.5
Ash, %DM	11.1	10.2

^1^ Contains per kilogram premix: 10,000,000 IU vitamin A; 70,000 IU vitamin E; 1,600,000 IU vitamin D; 50 g Fe; 40 g Zn; 40 g Mn; 0.1 g Co; 10 g Cu; 0.1 g Se; 0.5 g I

**Table 2:** Chemical compositions of rubber seed kernel pellet (RUSKEP)

Item	RUSKEP
Chemical composition	
Dry matter, %	90.1
Organic matter, %DM	94.9
Crude protein, %DM	19.8
Ether extract, %DM	27.3
Neutral detergent fiber, %DM	25.1
Acid detergent fiber, %DM	7.1
Ash, %DM	5.1
Gross energy, kcal/kg DM	8,162.2
Fatty acid, % of total fatty acid	
C16:1 cis-9	0.2
C18:0	6.4
C18:1 cis-9 + trans-9 (OA)	21.2
C18:2 cis-9,12 + trans-9,12 (LA)	49.7
C18:3 cis-9,12,15 (ALA)	13.4
C20:0	0.2
C20:1 cis-11 (EA)	0.2
C22:0	0.1

OA = oleic acid; LA = linoleic acid; ALA = α-linolenic acid; EA = eicosanoic acid

### Gas kinetics, cumulative gas production, and in vitro degradability

The gas kinetics and cumulative gas production were similar among treatments (*P* > 0.05) (Table 3). The supplementation of RUSKEP did not alter in vitro dry matter degradability (IVDMD) or in vitro organic matter degradability (IVOMD) (*P* > 0.05) (Table 4).

**Table 3:** Effect of rubber seed kernel pellet (RUSKEP) supplementation on gas kinetics and cumulative gas production (96 h)

RUSKEP(%)	Gas kinetics				Gas (96 h) (ml/0.5 g DM substrate)
	a	b	c	a + b	
0	-0.4	89.8	0.05	89.3	89.3
2	-0.7	89.5	0.04	88.9	86.3
4	-0.7	108.7	0.03	108.0	103.5
6	1.7	93.8	0.04	95.5	93.6
8	0.3	105.2	0.04	105.5	101.2
10	-1.2	95.8	0.03	94.6	82.8
SEM	1.14	9.61	0.005	9.26	8.52
Contrast					
Linear	0.88	0.45	0.07	0.42	0.97
Quadratic	0.28	0.39	0.62	0.31	0.16
Cubic	0.21	0.87	0.19	0.75	0.40

**Table 4:** Effect of rubber seed kernel pellet (RUSKEP) supplementation on degradability, ruminal pH and NH_3_-N

RUSKEP (%)	Degradability (%)		pH	NH_3_-N (mg/dl)
	IVDMD	IVOMD		
0	40.7	56.6	6.6	28.6
2	37.8	54.1	6.6	26.3
4	37.0	57.7	6.6	21.0
6	39.3	55.0	6.6	25.1
8	37.9	54.2	6.5	24.5
10	34.0	53.2	6.5	26.8
SEM	1.77	1.85	0.02	1.31
Contrast				
Linear	0.10	0.26	< 0.01	0.37
Quadratic	0.67	0.55	0.28	< 0.01
Cubic	0.21	0.82	0.92	0.47

IVDMD = in vitro dry matter degradability; IVOMD = in vitro organic matter degradability; NH_3_-N = Ammonia nitrogen; SEM = standard error of the mean

### Rumen fermentation

The ruminal pH decreased linearly (*P* < 0.01) when supplemented with RUSKEP (Table 4). Ruminal NH_3_-N decreased quadratically (*P* < 0.01) by RUSKEP supplementation. The proportions of C3, C4, valerate (C5), and iso-valerate (i-C5) increased linearly (*P* ≤ 0.01), whereas C2 and C2:C3 decreased linearly (*P* < 0.01) with the addition of RUSKEP (Table 5).

**Table 5:** Effect of rubber seed kernel pellet (RUSKEP) supplementation on VFA in the rumen

RUSKEP (%)	Total VFA (mmol/l)	VFA (mol/100 mol)						C2:C3
		C2	C3	C4	i-C4	C5	i-C5	
0	64.5	69.5	18.9	9.8	0.5	0.7	0.7	3.7
2	66.2	69.4	19.1	9.5	0.5	0.7	0.7	3.7
4	65.2	68.8	19.4	10.0	0.5	0.7	0.7	3.6
6	67.8	67.7	19.7	10.6	0.5	0.7	0.8	3.4
8	66.2	66.8	20.3	10.8	0.5	0.8	0.8	3.3
10	66.6	66.2	20.7	11.0	0.6	0.8	0.8	3.2
SEM	2.21	0.80	0.41	0.36	0.02	0.03	0.03	0.11
Contrast								
Linear	0.50	< 0.01	< 0.01	< 0.01	0.39	< 0.01	0.01	< 0.01
Quadratic	0.67	0.65	0.57	0.74	0.51	0.14	0.47	0.60
Cubic	0.99	0.59	0.89	0.28	0.07	0.31	0.62	0.67

C2 = acetate; C3 = propionate; C4 = butyrate; i-C4 = iso-butyrate; C5 = valerate; i-C5 = iso-valerate; C2:C3 = acetate to propionate ratio; SEM = standard error of the mean

### Rumen fatty acid profiles

Capric acid (C10:0), myristic acid (C14:0), tetradecenoic acid (C14:1 cis-9), pentadecanoic acid (C15:0), palmitic acid (C16:0), and palmitoleic acid (C16:1 cis-9) levels were all lower when RUSKEP was added (Table 6). The supplementation of RSK increased quadratically OA (*P* < 0.01) and increased linearly LA, ALA, or eicosanoic acid (EA; C20:1 cis-11) (*P* < 0.02). The concentrations of the UFA, MUFA, and PUFA were higher (*P* < 0.01), while the SFA was lower (*P* < 0.01) with increasing levels of RUSKEP.

**Table 6:** Effect of rubber seed kernel pellet (RUSKEP) supplementation on fatty acid profiles in the rumen

Items	RUSKEP (%)						SEM	Contrast		
	0	2	4	6	8	10		Linear	Quadratic	Cubic
Fatty acid, % of total fatty acid										
C8:0	0.9	0.8	0.8	0.7	0.5	0.6	0.16	0.08	0.52	0.59
C10:0	1.6	1.3	0.9	1.3	0.7	0.9	0.21	0.01	0.40	0.74
C12:0	0.8	0.8	0.9	0.7	0.9	0.6	0.19	0.68	0.62	0.89
C14:0	5.3	3.4	3.3	2.8	2.9	2.3	0.60	< 0.01	0.19	0.27
C14:1 cis-9	2.1	2.4	1.8	1.8	1.5	1.3	0.30	0.02	0.60	0.43
C15:0	3.8	3.1	3.0	2.6	2.5	2.3	0.31	< 0.01	0.32	0.76
C16:0	7.2	6.6	7.0	6.5	5.9	5.5	0.53	0.03	0.52	0.84
C16:1 cis-9	1.2	1.3	0.9	0.7	0.7	0.6	0.26	0.03	0.73	0.58
C17:0	1.9	1.4	1.4	1.4	1.2	0.8	0.11	< 0.01	0.94	0.01
C18:0	35.7	32.7	32.8	34.7	33.4	30.8	1.26	0.07	0.73	0.04
C18:1 cis-9 + trans-9 (OA)	7.8	12.2	15.6	17.0	17.7	19.9	0.75	< 0.01	< 0.01	0.12
C18:2 cis-9,12 + trans-9,12 (LA)	2.6	3.7	6.0	5.7	7.2	11.3	0.90	< 0.01	0.18	0.10
C18:3 cis-9,12,15 (ALA)	0.7	0.8	1.3	1.3	1.5	2.4	0.17	< 0.01	0.07	0.09
C20:0	1.4	1.7	1.4	1.3	1.3	1.0	0.13	0.01	0.27	0.53
C20:1 cis-11 (EA)	ND	0.2	0.3	0.3	0.7	0.5	0.19	0.02	0.56	0.63
C22:0	0.6	0.4	0.4	0.9	0.4	0.4	0.24	0.75	0.69	0.27
SFA	59.4	52.4	51.8	52.8	49.7	45.3	1.36	< 0.01	0.82	< 0.01
UFA	14.4	20.6	26.0	26.8	29.2	36.0	1.30	< 0.01	0.45	0.02
MUFA	11.1	16.1	18.7	19.8	20.6	22.3	0.88	< 0.01	0.01	0.11
PUFA	3.3	4.6	7.3	7.0	8.6	13.7	1.03	< 0.01	0.14	0.09

OA = oleic acid; LA = linoleic acid; ALA = α-linolenic acid; EA = eicosanoic acid; SFA = saturated fatty acids; UFA = unsaturated fatty acids; MUFA = mono-unsaturated fatty acids; PUFA = poly-unsaturated fatty acids; SEM = standard error of the mean

## Discussion

The amount of CP and EE in the RUSKEP was 19.8% and 27.3%, which was lower than in our previous evaluations [9–11]. This result could be due to the use of RSK as the major ingredient and the addition of minor ingredients, including cassava starch, molasses, a mineral and vitamin mixture, or salt, in the process of making the pellet, resulting in a dilution of the CP and EE contents in the RUSKEP. The GE content at 8,162.2 kcal/kg DM was similar to our previous research [10, 12]. In addition, RUSKEP was rich in UFA content, including OA, LA, and ALA, and was consistent with earlier studies [12]. This indicates that RUSKEP has the potential to be used as a protein, an energy source, and a C18 UFA.

The current study uses a roughage to concentrate ratio (R: C) of 70:30, which shows a lower IVDMD (34.0–40.7%) with an added RUSKEP, while our previous study used RSK in the diet with a R: C of 40:60 and found a higher IVDMD (55.8–57.7%) [12]. An excessive supply of nitrogen, which causes a release of ruminal NH_3_ that is incorporated into microbial proteins, led to the loss of a significant amount of nitrogen as NH_3_ that was absorbed from the rumen [18]. The ruminal NH_3_-N ranged from 21.0 to 28.6 mg/dL in the current study, which is closer to the optimal range (20 to 35 mg/dL) for rumen microorganisms, fermentation, and microbial protein synthesis [10, 19]. The addition of RUSKEP at 4% has led to a lower ruminal NH_3_-N concentration. Ruminal microbial protein synthesis is highly dependent on the availability of degradable carbohydrates and proteins [20]. More NH_3_-N gets incorporated into microbial protein when the ruminants are fed more non-fiber carbohydrate (NFC), which has been demonstrated to be the main source of energy for ruminal microbes [21]. The increase in dietary protein from RUSKEP at 4% and the use cassava chip as the main NFC in concentrate may contribute to microbial protein synthesis and reduce ruminal NH_3_-N. However, adding RUSKEP at 6–10% had no effect on ruminal NH_3_-N when compared to the control. It is possible that the energy level, especially NFC, is not enough to support microbial protein yields and higher ruminal NH_3_-N in our study.

Ruminal volatile fatty acids (VFA) production is important because VFA provides the energy source for the ruminant [22]. Ruminal concentrations of VFA have been utilized to characterize the progression of ruminal fermentation and the effects of dietary treatments [23]. Ruminants primarily use C3 as a precursor to glucose; therefore, increased C3 production may contribute to improved feed energy utilization and productivity efficiency [24]. Supplementation RUSKEP increased C3 and C4 while reducing C2, which resulted in a decreased C2:C3 ratio. Similary, Pi et al. [14] found that the proportion of ruminal C2 decreased and the proportion of C3 increased when supplemented with rubber seed oil in dairy cows; simultaneously, the C2:C3 ratio reduced. Dey et al. [25] reported that the addition of vegetable oils could improve in vitro rumen fermentation with a higher proportion of C3 and C4 and a lower C2:C3 ratio using rumen fluid from buffalo. There are two possible mechanisms that could explain the shift in VFA production. First, when the diet contains lipid, the lipolytic bacteria hydrolyze it into glycerol, which promptly ferments into VFA, particularly C3 and C4 [25]. Second, the addition of oilseeds rich in polyunsaturated fatty acids (PUFA), specifically LA and ALA, induces a detrimental impact on fibrolytic bacteria by impairing cell integrity [26, 27]. Hence, ruminal fermentation is shifting toward C3 as a result of these microbial alterations, while also decreasing C2 production. Previous reports of goats fed dietary oilseed rich in PUFA have indicated a decrease in the populations of *Fibrobacter succinogenes* and *Ruminococus flavefaciens*, the predominant fibrolytic bacteria in the rumen [28]. Supplementation of RUSKEP up to 10% may reduce the population of fibrolytic bacteria in the rumen, resulting in lower C2 and higher C3, then subsequently decreasing C2:C3.

The lipids of oilseeds used as feed for animals, such as flaxseed or rubber seed, are predominantly triglycerides containing OA, LA, and ALA [29]. Upon entering the rumen, microorganisms are capable of hydrogenating dietary UFA, and the final product is mainly steric acid (C18:0). This process, known as biohydrogenation, involves cis and trans isomerization to trans fatty acid intermediates, followed by hydrogenation of the double bonds [3]. LA is typically isomerized to CLA, which is then hydrogenated to VA. ALA is changed to C18:3 cis-9, trans-11, cis-15, and C18:2 trans-11, cis-15, and then hydrogenated to VA. Subsequently, VA is converted into C18:0 in the rumen. However, the incomplete conversion of unsaturated 18-carbon fatty acids (FA) to 18:0 leads to the accumulation of OA, LA, and ALA intermediates in the rumen, which are then absorbed and incorporated into their meat and milk [30]. In the present study, the biohydrogenation of C18 FA was lower with RUSKEP supplementation. The supplementation with RUSKEP, up to 10%, enhanced C18 UFA, including OA, LA, and ALA in the rumen. This result is consistent with a previous study that indicated that rubber seed oil and flaxseed oil supplementation increased the levels of OA and ALA in the rumen of dairy cows [14]. The supplementation with oilseed reduced in vitro rumen biohydrogenation in the rumen, enhancing C18 UFA and reducing SFA in the rumen [31, 32]. Alterations in ruminal biohydrogenation and FA composition of rumen fluid in response to the addition of oilseeds were divided into two groups: biohydrogenation of UFA from the oilseed directly and the influence of FA in oilseed on various dietary UFA [33].

Pelleting is a mechanical process that utilizes heat, moisture, and pressure to combine and incorporate feed into pellets. Heat treatment of oilseed is used to reduce the biohydrogenation of C18 UFA in the rumen [34]. We found that adding RSK pellets up to 10% reduced the rumen biohydrogenation of OA, LA, and ALA. Similarly, adding hot air oven-heated rubber seed kernels to the in vitro study’s findings resulted in reduced biohydrogenation in the rumen and increased levels of OA, LA, and ALA in our previous research [12]. These findings may be due to the heat process during the RSK pellet, which could have denaturated the protein structure around fat droplets, forming an inhibitory encapsulation against biohydrogenation in the rumen [35]. Another possibility is that the heat process of the oilseed pellet may be attributed to a decrease in lipolysis in the rumen as an effect of protecting lipid droplets [36]. This indicated that RUSKEP inhibited the complete hydrogenation of C18 UFA to C18:0 in the rumen, leading to C18 UFA (OA, LA, and ALA) accumulating in the rumen fluid of swamp buffalo. Moreover, there has been an accumulation of some biohydrogenation intermediates of very long-chain PUFA with 20 carbons [3]. The addition of RUSKEP increased EA in the rumen. Similarly, Pi et al. [14] reported that the supplementation of rubber seed oil in dairy cows increased EA in the rumen. These results indicated that RUSKEP supplementation enhanced the ruminal concentration of C18 MUFA and PUFA with reduced SFA in swamp buffalo rumen.

## Conclusions

Supplementing RUSKEP up to 10% of the diet was a strategy to improve fermentation parameters and C18 UFA production, especially ALA, in the rumen. More research is required to determine the effects of RUSKEP supplementation on feed intake, digestibility, rumen fermentation, and meat quality in swamp buffalo.

## Materials and methods

### Ethical Procedure

The authors confirmed that all methods were carried out in accordance with the applicable guidelines and regulations. The Animals Ethical Committee of Rajamangala University of Technology Isan approved all of the experimental animals and methodology used in this study (approval number 04-06-004). The study was carried out in compliance with the ARRIVE guidelines.

### Preparation of RUSKEP

Experimental research and field studies on plants (either cultivated or wild), including the collection of plant material, must comply with relevant institutional, national, and international guidelines and legislation. The *Hevea brasiliensis* (rubber) tree belongs to the family Euphorbiaceae. Rubber seeds were purchased from the local markets in Sakon Nakhon, Thailand, during the harvest season. The seeds were collected by hand from the ground and stored indoors. A dehulling machine (Incanewlife, Khon Kaen, Thailand) removed the shells from the seeds. The kernels were dried in sunlight for three days, followed by grinding to pass through a 1 mm sieve. The RUSKEP was prepared by using RSK as a major ingredient and other ingredients such as cassava starch, molasses, a mineral and vitamin mixture, or salt, and then making the mixture into pellets using a pellet machine.

### Experimental design and dietary treatments

This study used a CRD to compare the supplementation of RUSKEP at 0, 2, 4, 6, 8, and 10% of DM substrates. The diet had a R: C of 70:30, and roughage was obtained by the use of rice straw. Analyses were conducted on the samples to assess the amount of DM, ash, EE, CP [37]. The neutral detergent fiber (NDF) and acid detergent fiber (ADF) using a fiber analyzer (ANKOM 200, ANKOM Technology, NY, USA). The GE value of a feed was estimated with bomb calorimetry (Oxygen Bomb Calorimeter; Parr Instrument Company, Moline, IL, USA). The gas chromatography (GC 8890; Agilent Technologies Ltd., Santa Clara County, CA, USA) analysis of fatty acid profiles using the method of Cristie [38].

### Animals and preparation of rumen inoculum

The animals were used and carried out at the farm of the Faculty of Natural Resources, Rajamangala University of Technology Isan, Sakon Nakhon Campus, Phangkhon, Sakon Nakhon, Thailand. Two male swamp buffaloes with a body weight (BW) of 260 ± 10 kg were used to provide rumen fluid. Concentrate has been added at 1% of BW, and rice straw was offered *ad libitum* for a period of 14 days to the buffalo. The animals were fed twice a day, at 7:00 and 16:00 h. Before morning feeding on day 15, 1,000 mL of rumen fluid was taken from each animal using a stomach tube connected to a vacuum pump. Four layers of cheesecloth were used to filter the rumen fluid, which was then put into pre-heated thermos flasks before it was transferred to the lab [39].

The 0.5 g of feed sample was weighed into 50-mL bottles, and RUSKEP was added at 0, 2, 4, 6, 8, and 10%. There were four replicates of each treatment, and there were 24 sample bottles and 4 blanks, for a total of 28 bottles. The ruminal fluid from each buffalo had been mixed with the artificial saliva solution in a ratio of 2:1 (mL/mL) at 39 °C with continuous CO_2_ flushing [40]. Each bottle had 40 mL of rumen inocula mixture, and it was flushed with CO_2_. The bottles have been covered with aluminum caps and rubber stoppers. They were shaken at 60 rpm and incubated at 39 °C (Stuart orbital incubator S1600, Staffordshire, UK).

### In vitro gas production and fermentation characteristics

At 0, 2, 4, 6, 8, 12, 24, 48, 72, and 96 h, the volume of gas produced was recorded using a 25 mL calibrated glass syringe with an air connector. Four bottles containing only rumen inoculums were included in each run for each sample time, and the average gas production amounts from these bottles were employed as blanks. The net volume of gas was determined by subtracting the blank values from each obtained value. The gas production data were fitted to the model of Ørskov and McDonald [41] as follows: y = a + b [1 − e^(− ct)^], where a = the gas production from the immediately soluble fraction, b = the gas production from the insoluble fraction, c = constant rate of gas production for the insoluble fraction, a + b the potential extent of gas production, t = incubation time, and y = gas production at time “t”.

At 24 h of incubation, IVDMD and IVOMD [42] were evaluated using 24 bottles (4 bottles per treatment, 6 treatments). The pH (FiveGo, Mettler-Toledo GmbH, Greifensee, Switzerland), NH_3_-N (Kjeltech Auto 1030 Analyzer, Tecator, Hoganiis, Sweden) [43], VFA [44], and fatty acid profiles [38] (GC 8890; Agilent Technologies Ltd., Santa Clara County, CA, USA) of ruminal fluid were evaluated using a second set of 24 bottles (4 bottles per treatment, 6 treatments).

### Statistical analysis

The SAS program was used to analyze all data, and the GLM procedure was utilized to conduct the analysis of variance [45]. The data were examined using the following model: Yij = *µ* + αi + εij, where Yij is the observation, *µ* is the overall mean, αi is the treatment effect, and εij is the residual error. Considered significant were statistical treatment parameter differences with a *P* < 0.05. Using orthogonal polynomial contrasts (linear, quadratic, and cubic), the treatment trends were statistically assessed.

## Data Availability

The datasets obtained and analyzed during the present study are available upon reasonable request from the corresponding author.

## Abbreviations

UFA: Unsaturated fatty acids
RUSKEP: Rubber seed kernel pellet
CRD: Completely randomized design
DM: Dry matter
NH_3_-N: Ammonia-nitrogen
C2: Acetate
C3: Propionate
C4: Butyrate
C2:C3: Acetate to propionate ratio
OA: Oleic acid
LA: Linoleic acid
ALA: α-linolenic acid
SFA: Saturated fatty acids
MUFA: Mono-unsaturated fatty acids
PUFA: Poly-unsaturated fatty acids
RSK: Rubber seed kernel
CP: Crude protein
EE: Ether extract
GE: Gross energy
VA: Vaccenic acid
CLA: Conjugated linoleic acid
IVDMD: In vitro dry matter degradability
IVOMD: In vitro organic matter degradability
C5: Valerate
i-C5: Iso-valerate
EA: Eicosanoic acid
NFC: Non-fiber carbohydrate
VFA: Volatile fatty acids
FA: Fatty acids
NDF: Neutral detergent fiber
ADF: Acid detergent fiber
BW: Body weight
R:C: Roughage to concentrate ratio
